# Activating Autophagy Enhanced the Antitumor Effect of Antibody Drug Conjugates Rituximab-Monomethyl Auristatin E

**DOI:** 10.3389/fimmu.2018.01799

**Published:** 2018-08-03

**Authors:** Yichen Wang, Xuyao Zhang, Jiajun Fan, Wei Chen, Jingyun Luan, Yanyang Nan, Shaofei Wang, Qicheng Chen, Yujie Zhang, Youling Wu, Dianwen Ju

**Affiliations:** ^1^Department of Microbiological and Biochemical Pharmacy, The Key Laboratory of Smart Drug Delivery, Ministry of Education, School of Pharmacy, Fudan University, Shanghai, China; ^2^Zhejiang Teruisi Pharmaceutical Co. Ltd., Huzhou, Zhejiang, China

**Keywords:** non-Hodgkin lymphoma, antibody drug conjugates, autophagy, apoptosis, combination therapy

## Abstract

**Background:**

Antibody drug conjugate (ADC) showed potent therapeutic efficacy in several types of cancers. The role of autophagy in antitumor effects of ADC remains unclear.

**Methods:**

In this study, the ADC, Rituximab-monomethyl auristatin E (MMAE) with a Valine–Citrulline cleavable linker, was designed to investigate its therapeutic efficacy against non-Hodgkin lymphoma (NHL) as well as the underlying mechanisms. Methylthiazolyldiphenyl-tetrazolium bromide (MTT) was used to detect growth inhibition in B-cell lymphoma cell lines, Ramos and Daudi cells, which were treated by Rituximab-MMAE alone or combined with autophagy conditioner. Apoptosis was detected by flow cytometry and immunohistochemistry, and apoptosis inhibitor was employed to discover the relationship between autophagy and apoptosis during the Rituximab-MMAE treatment. Autophagy was determined by three standard techniques which included confocal microscope, transmission electron microscope, and western blots. Ramos xenograft tumors in BALB/c nude mice were established to investigate the antitumor effect of combination use of Rituximab-MMAE and autophagy conditioner in B-NHL therapy.

**Results:**

Our results showed that Rituximab-MMAE elicited caspase-3-dependent apoptosis in NHL cells and exhibited potent therapeutic efficacy *in vivo*. Autophagy, which was characterized by upregulated light chain 3-II expression, and accumulation of autophagosomes, was triggered during the Rituximab-MMAE treatment. Meanwhile, inactivation of Akt/mTOR pathway was shown to be involved in the autophagy triggered by Rituximab-MMAE, indicating a probable mechanism of the ADC-initiated autophagy. Importantly, inhibition of autophagy by chloroquine suppressed the Rituximab-MMAE-induced apoptosis, while activating autophagy by rapamycin significantly enhanced the therapeutic effect of Rituximab-MMAE both *in vitro* and *in vivo*.

**Conclusion:**

Our data elucidated the critical relationship between autophagy and apoptosis in Rituximab-MMAE-based therapy and highlighted the potential approach for NHL therapy by combined administration of the ADC and autophagy activator.

## Introduction

Non-Hodgkin lymphoma (NHL) is one of the most common types of lymphomas which accounts for about 90% of all cases (1). Overall survival rate of NHL has dramatically improved in decades due to the application of anti-CD20 monoclonal antibodies, such as Rituximab, and refined chemotherapy regimens, but relapse and resistance have raised a big challenge in the treatment of NHL (2–4). Thus, novel therapeutic approaches for NHL are urgently needed.

As one of the most promising target therapy strategies, antibody drug conjugate (ADC) was characterized by monoclonal antibody conjugated with small molecule drugs, which has shown significant therapeutic efficacy in the treatment of hematological malignancies and solid tumors (5). By covalent conjugated to monoclonal antibody, highly potent cytotoxic agents could be delivered to tumors which minimize toxicity to healthy tissue (6). Despite the concept of ADC is straightforward, the early failures in development of ADC due to high systemic toxicity and therapy resistance suggested more unknown mechanism need to be dug out (7, 8).

Autophagy is a homeostatic process in eukaryotic cells which degrades intracellular components for energy recycling and reusing (9). Growing evidence showed that autophagy acted as a “double-edged sword” in cancer therapy (10). Some studies reported that upregulated autophagy was observed in cancer cells to maintain the aggressiveness and protect tumor cells from anticancer therapy, while autophagy served as a cell death mechanism for type II programmed cell death under certain cell circumstances (11, 12). It was reported that arsenic trioxide and vitamin D combined with radiation therapy lead to a cytotoxic autophagy in leukemia and breast cancer cells, respectively (13, 14). Furthermore, highly toxic materials such as poly amidoamine dendrimers, quantum dots, and silicon nanoparticles could trigger cytotoxic autophagy and finally led to multiple organ disorders (15–18). Although autophagy plays an important and complicated role in cancer therapy, the relationship between autophagy and ADC’s treatment is still unclear.

In this study, the ADC, Rituximab-monomethyl auristatin E (MMAE), was designed and taken into practice to show potent therapeutic effects both *in vitro* and *in vivo*. It was noteworthy that autophagy was activated and demonstrated to play crucial roles in Rituximab-MMAE-mediated therapeutic effects. Combined administration of ADC and autophagy activator rapamycin significantly potentiated the therapeutic effects of Rituximab-MMAE, implying that activation of autophagy might be a novel approach to improve the antitumor efficacy of the ADC.

## Materials and Methods

### Cell Culture

Human NHL Ramos and Daudi cells were purchased from the Type Culture Collection of the Chinese Academy of Sciences, Shanghai, China. Ramos and Daudi cells were cultured in RPMI-1640 medium supplemented with 10% fetal bovine serum, 100 U/mL penicillin, and 100 µg/mL streptomycin, and the cells were incubated at 37°C in humidified air with 5% CO_2_.

### Preparation of Rituximab-MMAE

The reduction of anti-CD20 antibody (Rituximab, produced by Teruisi Pharmaceuticals, Huzhou, China) for conjugation purposes was performed as previously described (19, 20). Briefly, the antibody was added into sodium phosphate buffer, followed by mixing with Tris(2-chloroethyl) phosphate (Sigma). MMAE linked with valine–citrulline linker was added slowly to the pre-chilled and reduced antibody solution for conjugation reaction. The drug antibody ratio (DAR) was determined by hydrophobic interaction chromatography. The purity of Rituximab-MMAE was above 95%, the average DAR of Rituximab-MMAE was 4.2 ± 0.5, and the content of endotoxin was below 0.3 EU/mg. The structure and chemical characteristic of ADC can be found in Figures S1 and S2 in Supplementary Material.

### Cell Viability Assay

Cell viability of the NHL cells was determined by methylthiazolyldiphenyl-tetrazolium bromide (MTT)-based assay. Ramos and Daudi cells were seeded into 96-well plates and treated by Rituximab-MMAE and autophagy inhibitor chloroquine or autophagy activator rapamycin After 48 h of co-incubation, MTT (0.5 mg/mL) was added into the wells and cells were maintained in the humidified incubator at 37°C for 4 h. Then, 200 µL of heated lysis solution (20% SDS, 50% DMF) was added in each well to dissolve the product formazan. The number of surviving cells was reflected by the absorbance at wavelength 570 nm, cell viability was expressed as the percentage of the absorbance of the control.

### Transmission Electron Microscope

Ramos and Daudi cells were incubated with or without Rituximab-MMAE for 24 h, cells were harvested and prepared according to the described process (18), samples were detected with a JEM 1410 transmission electron microscope (TEM; JEOL, Inc.).

### Western Blot

The total protein of Ramos and Daudi cells, including light chain 3 (LC3), SQSTM1, Caspase 3, Caspase 9, poly ADP-ribose polymerase (PARP), Bax, Bcl-2, p-mTOR, p-Akt, p-P70S6K, p-4E-BP-1, and β-actin, were homogenized in RIPA lysis buffer (Beyotime Biotechnology, Haimen, China) for 30 min on ice. The lysate was centrifuged at 12,000 rpm for 10 min at 4°C. Equivalent amount protein in each sample was separated in SDS-PAGE gel and electro-transferred onto polyvinylidene fluoride membranes. PVDF membranes were incubated with primary antibody and second antibody, respectively. Immobilon™ Western Chemiluminescent HRP Substrate (Millipore, Billerica, MA, USA) was used to visualize the immunoreactive bands, and densitometric values were quantified by ImageJ 1.47v (National Institutes of Health, USA).

### Confocal Microscope

Ramos and Daudi cells were treated with 40 ng/mL of Rituximab-MMAE for 24 h. Afterward, the cells were stained by Cyto-ID Autophagy Detection Kit (ENZO Life Science, Farmingdale, NY, USA) following the manufacturer’s instruction. Cells were then incubated with autophagy inducer, rapamycin (50 nM) for positive control and observed by inverted confocal microscope (Carl Zeiss LSM710, Carl Zeiss, Germany).

### Apoptosis Analysis

The Annexin V-FITC/PI Apoptosis Detection Kit (BD Biosciences, San Diego, CA, USA) was applied for apoptosis detection of Ramos and Daudi cells according to the manufacturer’s instructions. The analysis was performed on FACSCalibur flow cytometer (Becton-Dickinson, Fullerton, CA, USA). Thirty thousand cells per sample were analyzed.

### Xenograft Model

The NHL xenograft models were established in BALB/c nude mice. Six-week-old male BALB/c nude mice were injected with 1 × 10^7^ of Ramos cells subcutaneously, and cells were suspended in RPMI-1640 medium and 50% Matrigel Matrix (Coining, 354234). The tumor-bearing mice were divided into the indicated groups randomly, Rituximab-MMAE was intravenously injected twice a week while chloroquine and rapamycin were intraperitoneal injected once a day. All procedures involving animals were conducted in accordance with the protocols approved by Animal Ethical Committee of School of Pharmacy Fudan University.

### Statistical Analysis

GraphPad Prism 5 (GraphPad Software Inc., San Diego, CA, USA) was used to analyze the data. The results were expressed as means ± SD. Comparisons were performed using Student’s *t*-test (two-tailed) and one-way ANOVA. *P* value < 0.05 was considered statistically significant.

## Results

### Rituximab-MMAE Showed Potent Antitumor Effects *In Vitro* and *In Vivo*

**Figure 1:**
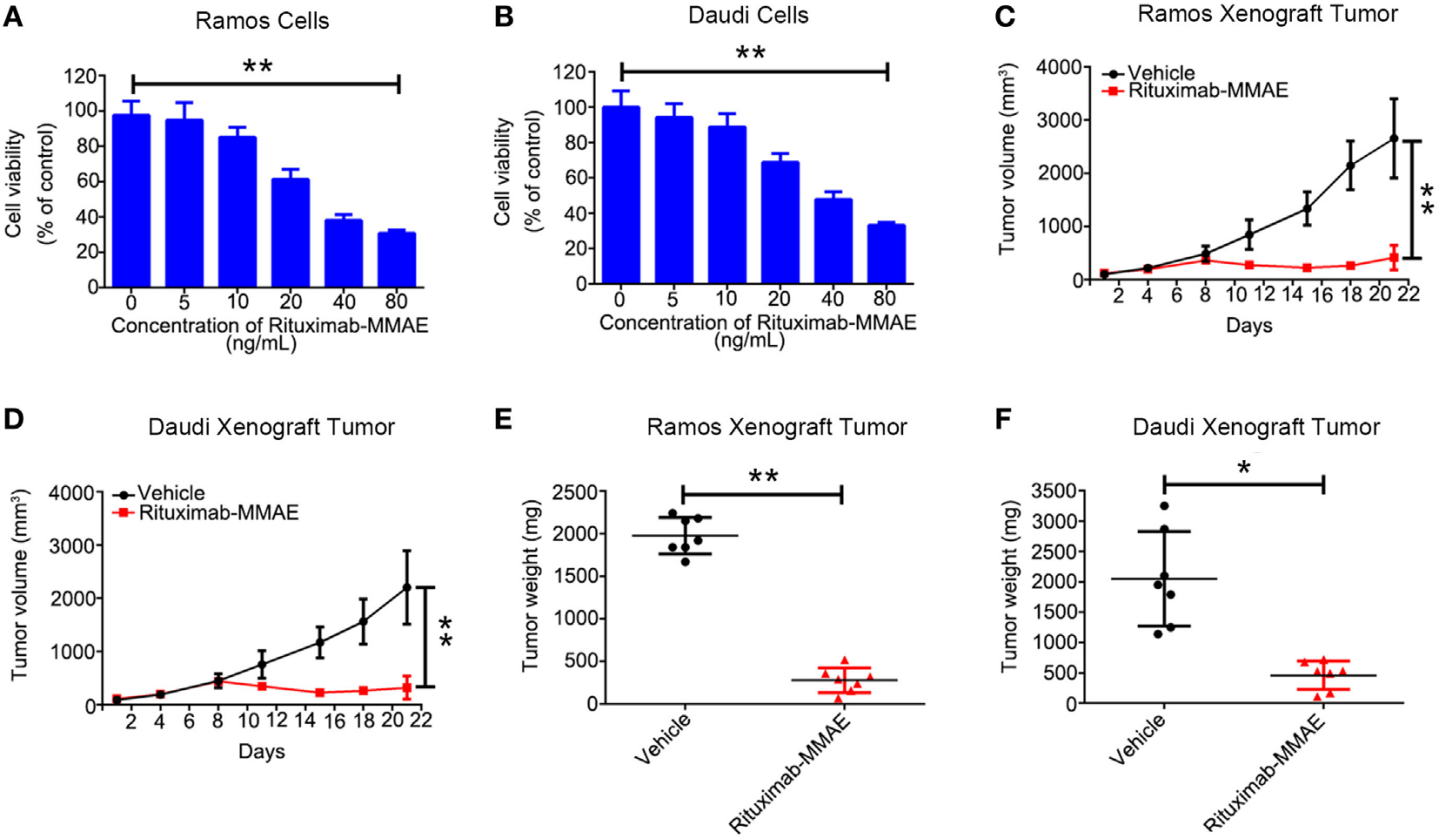
Rituximab-monomethyl auristatin E (MMAE) showed a potent antitumor effect *in vitro* and *in vivo*. **(A,B)** MTT assay was used to detect cytotoxicity of Rituximab-MMAE in CD20 positive cells Ramos and Daudi cells. The data were presented as mean ± SD of three independent experiments (Student’s *t*-test, *P < 0.05 and **P < 0.01). **(C,D)** BABL/c nude mice were transplanted subcutaneously with Ramos cells and Daudi cells. Xenograft tumor volume was evaluated every other day by direct caliper measurements. There are seven mice in each group. The data were presented as means ± SD. **(E,F)** After treatment with Rituximab-MMAE (0.75 mg/Kg) twice a week for 21 days, mice were sacrificed. Tumor weight was presented as mean ± SD, and each point represented a value from an independent mouse.

First, we found Rituximab-MMAE could be internalized by NHL cells rapidly (Figure S3 in Supplementary Material). Then, we investigated the cytotoxicity of Rituximab-MMAE against NHL cells *in vitro*. A dose-dependent cytotoxicity was observed in Ramos and Daudi cells after being exposed to Rituximab-MMAE (Figures 1A,B). Then, Ramos and Daudi xenograft models were established to further explore the anti-NHL efficacy of Rituximab-MMAE *in vivo*. Tumor growth was significantly inhibited from day 8 of Rituximab-MMAE therapy (Figures 1C,D). Mice were sacrificed on day 22 and the tumor weight in mice treated with Rituximab-MMAE, and the vehicle controls were 280.00 ± 144.80 and 1,977.14 ± 214.22 mg, respectively. Similarly, tumor weight in Daudi cell xenograft mice treated with Rituximab-MMAE was 460.00 ± 235.23 versus 2,050.00 ± 295.20 mg of the vehicle controls (Figures 1E,F). In addition, MMAE has limited influence on inhibition of tumor growth, and treatment with MMAE failed to enhance inhibitory efficacy of Rituximab on tumor growth. Rituximab-MMAE had a greater antitumor efficiency compared with Rituximab or Rituximab in combination with MMAE at the same dose (Figure S4 in Supplementary Material). Besides, our data showed that Rituximab-MMAE did not induce significant toxicity in the organs, indicating Rituximab-MMAE is safe for animal studies (Figure S5 in Supplementary Material). These data showed that Rituximab-MMAE had potent therapeutic efficacy both *in vitro* and *in vivo*.

### Rituximab-MMAE Induced Apoptosis in NHL Cells

**Figure 2:**
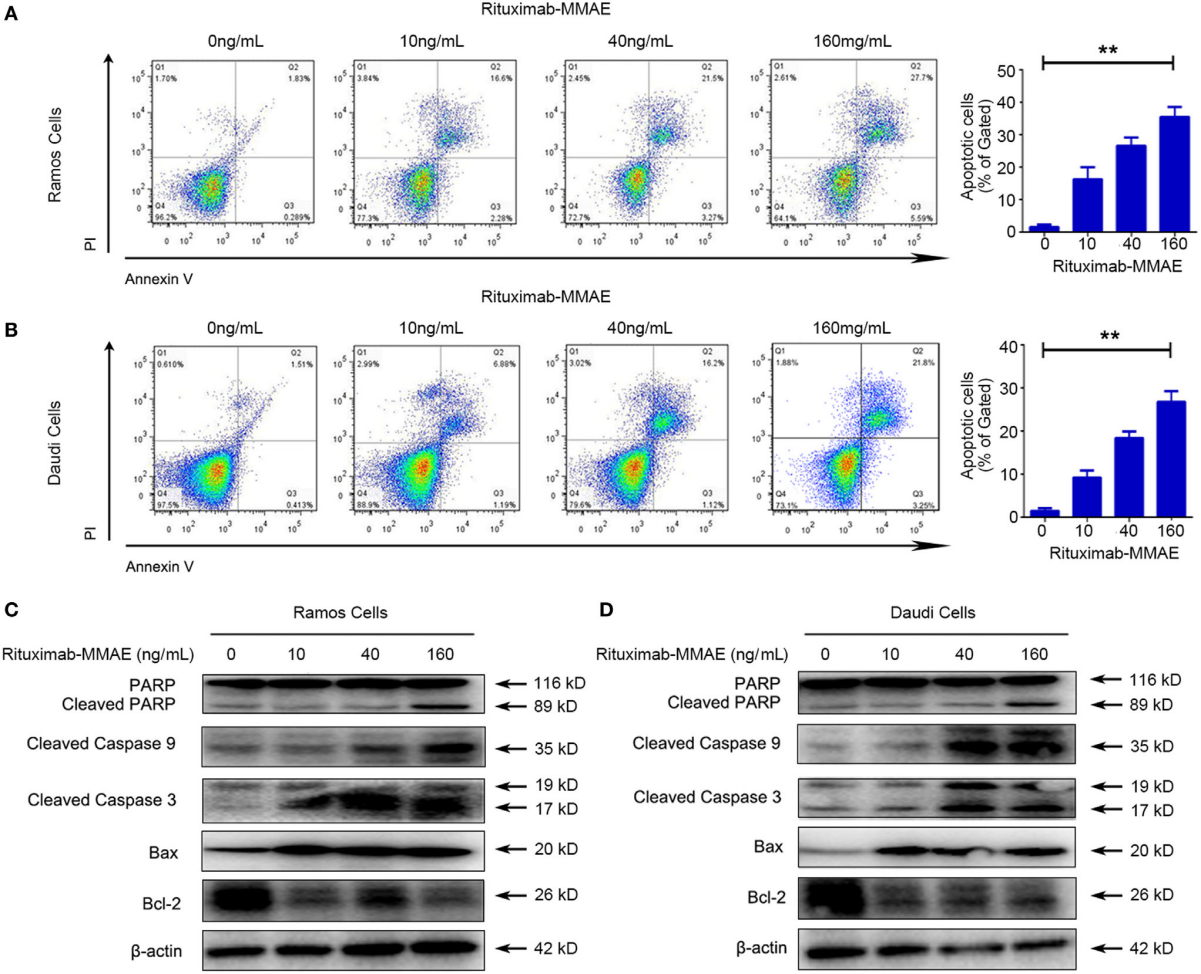
Rituximab-monomethyl auristatin E (MMAE) induced apoptosis in non-Hodgkin lymphoma cells. Apoptosis induced by Rituximab-MMAE was detected by western blot and flow cytometry (FCM). Ramos and Daudi cells were incubated in different concentration of Rituximab-MMAE. **(A,B)** Apoptosis was detected by FCM. The percentage of Annexin V+ PI− and Annexin V+ PI+ cells (apoptosis cells) was shown in right bar chart. **(C,D)** The protein level of poly ADP-ribose polymerase (PARP), cleaved PARP, cleaved caspase 9, cleaved caspase 3, Bax, and Bcl-2 was detected by western blot; β-actin was used as a loading control. Densitometric values were quantified by Image J software.

Secondly, flow cytometry (FCM) was applied to investigate whether apoptosis was triggered by Rituximab-MMAE. Figures 2A,B showed that treating with Rituximab-MMAE resulted in the increase of Annexin V positive cells in a dose-dependent manner in Ramos and Daudi cells, indicating that apoptosis was triggered by Rituximab-MMAE. Meanwhile, the hallmarks of classic apoptosis including activation of caspases and cleavage of PARP were detected. The results showed that caspase 9/3 and PARP were activated in Ramos cells and Daudi cells after treatment with Rituximab-MMAE (Figures 2C,D; Figure S6A in Supplementary Material). Furthermore, our result also demonstrated that Rituximab-MMAE downregulated the expression of Bcl-2 while upregulated the expression of Bax (Figures 2C,D; Figure S6E in Supplementary Material). Taken together, these data indicated that Rituximab-MMAE accelerated apoptosis in NHL cells.

### Rituximab-MMAE Triggered Formation and Accumulation of Autophagosomes

**Figure 3:**
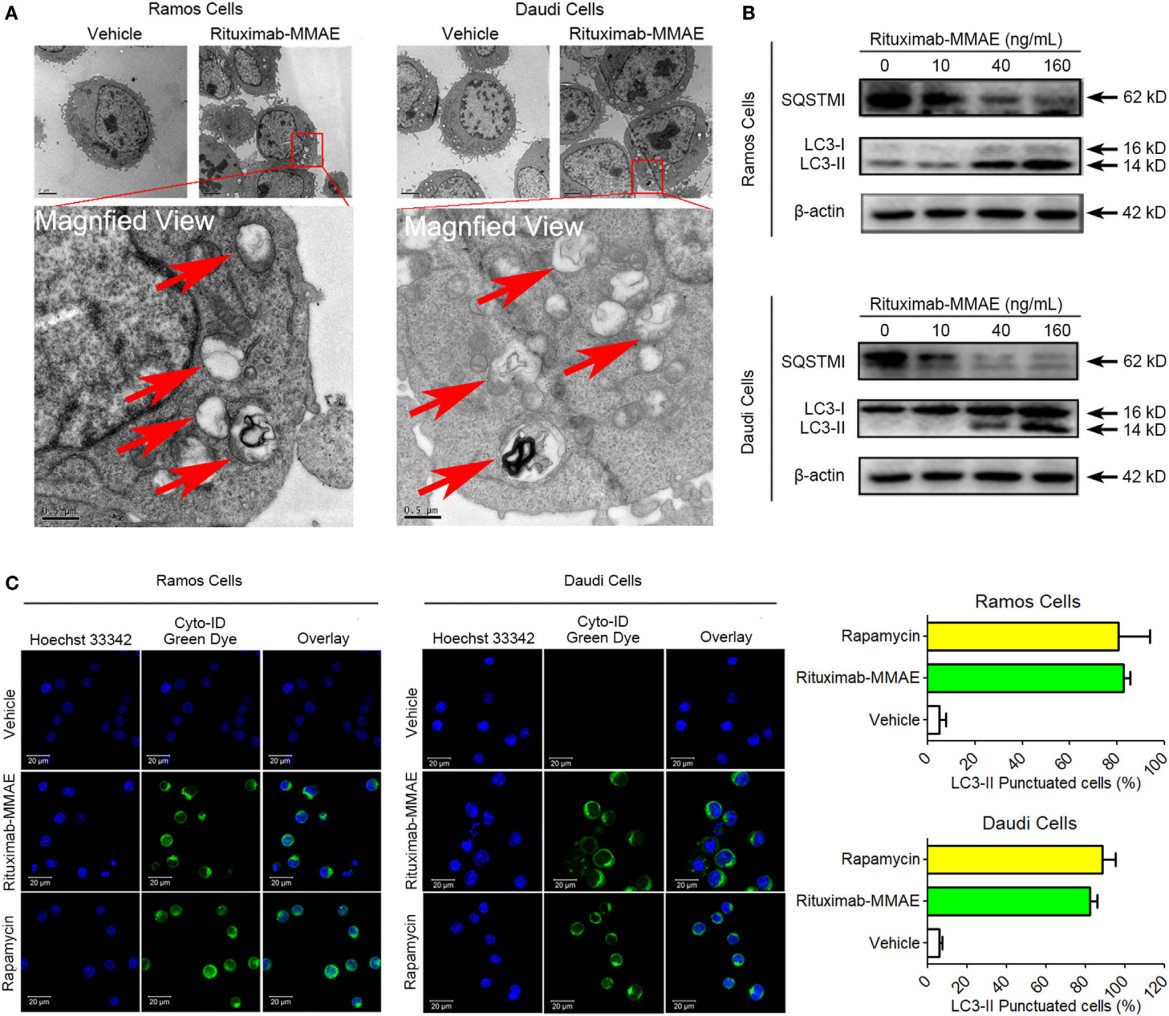
Rituximab-monomethyl auristatin E (MMAE) treatment induced accumulation of autophagosomes. **(A)** Ultrastructural analysis of Ramos and Daudi cells treated with Rituximab-MMAE. **(B)** The protein level of light chain 3 (LC3)-II and SQSTM1, two autophagic markers, in Ramos and Daudi cells after treatment with Rituximab-MMAE for 24 h. β-Actin was used as a loading control. Densitometric values were quantified by Image J software. **(C)** Extensive accumulations of autophagosomes in Ramos and Daudi cells were detected by Cyto-ID staining; Rapamycin was acted as the positive control. Results were presented as means ± SD of three independent experiments (Student’s *t*-test, *P < 0.05 and **P < 0.01).

To clarify the underlying mechanism of cytotoxicity and apoptosis elicited by Rituximab-MMAE, we investigated subsequently whether autophagy was activated in Rituximab-MMAE treatment. TEM analysis showed an abnormal formation and accumulation of autophagosomes, characterized by double-membrane vesicles, could be observed in the cytoplasm of Rituximab-MMAE-treated NHL cells as well as the tumor tissue (Figure 3A; Figure S7 in Supplementary Material). Furthermore, the autophagy initiation was confirmed by autophagy-related protein microtubule-associated protein 1 LC3 and sequestosome 1 (SQSTM1) expression. Western blots showed a significant decrease in the expression of SQSTM1 and increase of LC3-II protein in Ramos cells and Daudi cells in a dose-dependent manner after treatment with Rituximab-MMAE (Figure 3B; Figure S6B in Supplementary Material). In addition, Cyto-ID, a green dye for selectively labeling autophagic vacuoles, was used to determine the autophagosomes in Ramos and Daudi cells after treatment with Rituximab-MMAE. Similar to the positive control, the rapamycin-treated cells, cells exposed to Rituximab-MMAE showed a significantly punctate fluorescence increase, indicating autophagic vacuoles in the cytoplasm, which further confirmed the onset of autophagy (Figure 3C).

Therefore, our results demonstrated that Rituximab-MMAE significantly triggered autophagy in Ramos and Daudi cells.

### Rituximab-MMAE Inactivated the Akt/mTOR Signal Pathway

**Figure 4:**
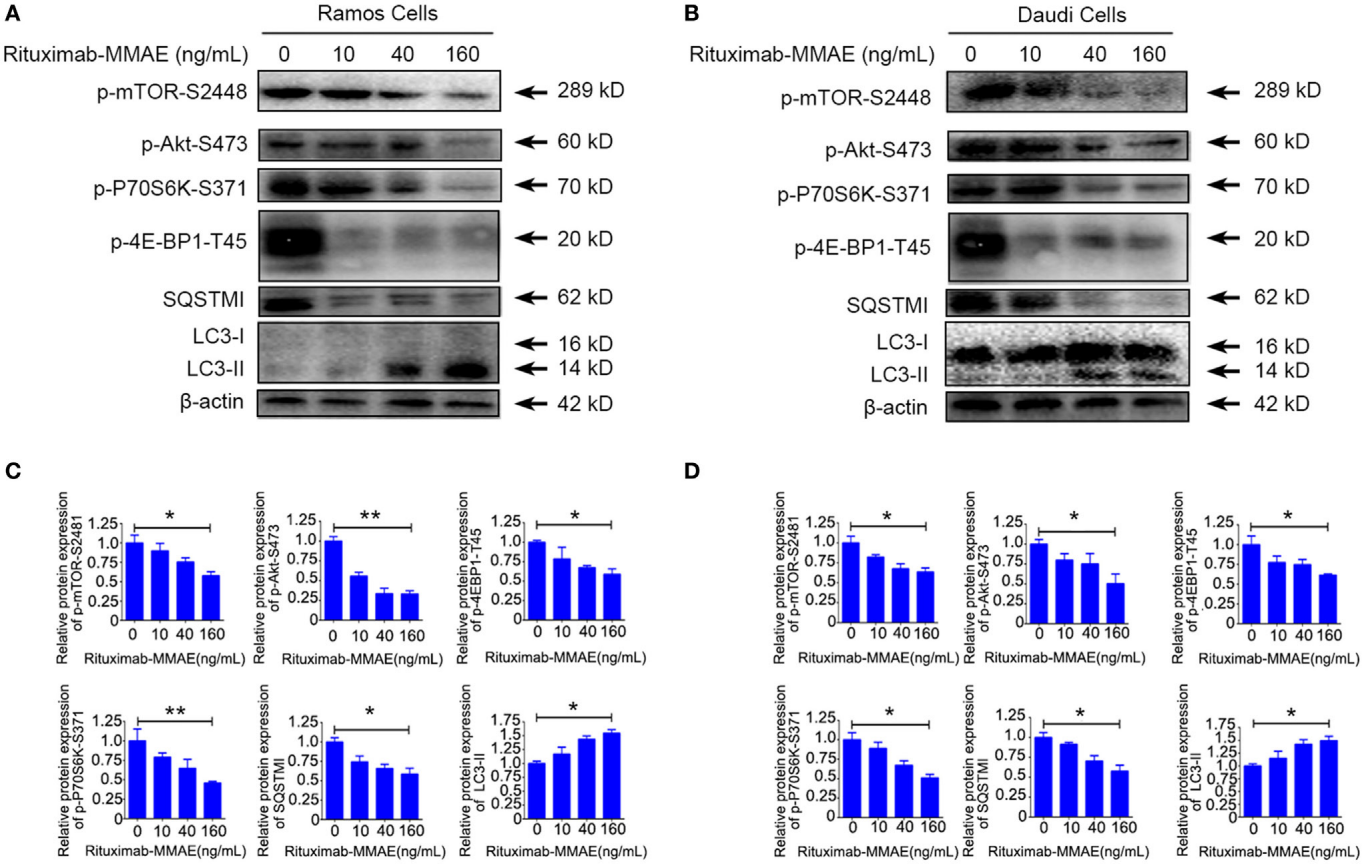
Inactivation of Akt/mTOR signal pathway induced by Rituximab-monomethyl auristatin E (MMAE) treatment. **(A,B)** Ramos cells and Daudi cells were exposed to various concentration of Rituximab-MMAE for 24 h, and whole-cell lysates were analyzed by western blot to examine the expression of SQSTM1, LC3, p-Akt, p-mTOR, p-P70S6K, and p-4E-BP1. **(C,D)** Densitometric values were quantified by Image J software and normalized to control. The values of control were set to 1.0. The data were presented as means ± SD of three independent experiments (Student’s *t*-test, *P < 0.05 and **P < 0.01).

To investigate the intracellular mechanism of autophagy induced by Rituximab-MMAE in Ramos cells and Daudi cells, the autophagy-related Akt/mTOR signaling pathway was detected in this study. Rituximab-MMAE decreased the amount of phosphorylated mTOR in a dose-dependent manner in Ramos cells and Daudi cells (Figures 4A,B). Besides, the phosphorylation of Akt, an upstream inducer of mTOR, was examined, and the result showed that the phosphorylation of Akt was efficiently inhibited. Moreover, phosphorylation of p70S6K and 4E-BP1, two downstream substrates of mTOR, was significantly inhibited in Ramos cells after treatment with Rituximab-MMAE (Figures 4A,B). The densitometric values of related protein were shown in Figures 4C,D. Thus, the signaling cascade exploration indicated that Rituximab-MMAE could inhibit Akt/mTOR signaling pathway which finally induced autophagy in NHL cells.

### Cytotoxicity of Rituximab-MMAE Could Be Modulated by Tuning Autophagy

**Figure 5:**
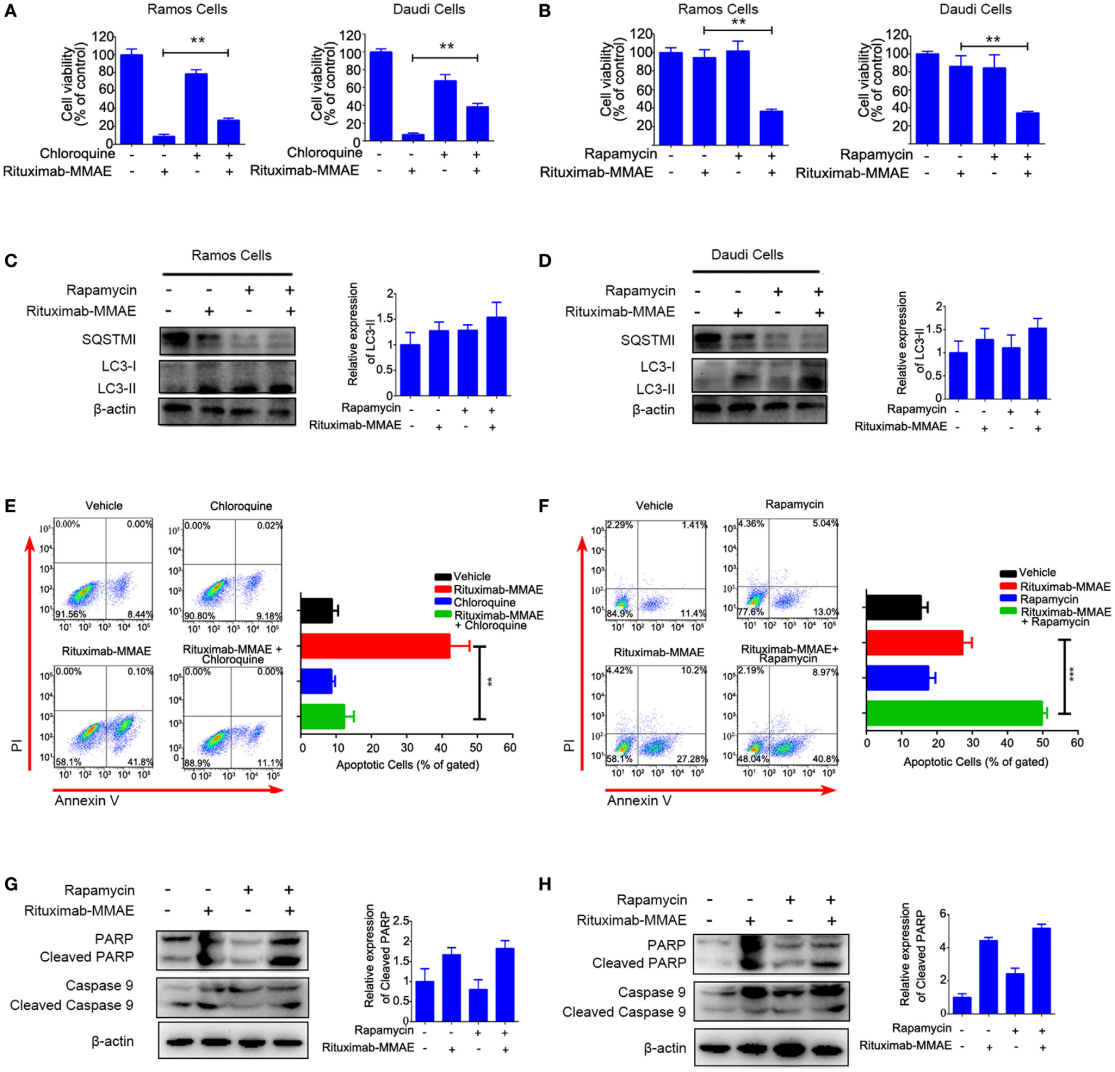
Autophagy involved in Rituximab-monomethyl auristatin E (MMAE)-induced cytotoxicity against non-Hodgkin lymphoma cells Ramos and Daudi. **(A,B)** MTT assay was used to detect cell viability of Ramos cells and Daudi cells treated with Rituximab-MMAE, with or without autophagy activator rapamycin (Rituximab-MMAE 40 ng/mL), or with or without autophagy inhibitor chloroquine (Rituximab-MMAE 160 ng/mL). The data were presented as mean ± SD of three independent experiments (Student’s *t*-test, *P < 0.05 and **P < 0.01). **(C,D)** The protein level of autophagic markers, SQSTM1 and light chain 3 (LC3)-II, were detected by western blot. Densitometric values were quantified by Image J software. **(E,F)** Apoptosis of Daudi cells treated with Rituximab-MMAE, with or without autophagy activator rapamycin, or with or without autophagy inhibitor chloroquine was detected by Annexin V and PI staining and flow cytometry. The percentage of Annexin V+ PI− and Annexin V+ PI+ cells (apoptosis cells) was shown in the bar chart on right. **(G,H)** Apoptosis protein, poly ADP-ribose polymerase (PARP), cleaved PARP, caspase 9, and cleaved caspase 9 were detected by western blot. Densitometric values were quantified by Image J software.

To explore the contribution of autophagy to cytotoxicity induced by Rituximab-MMAE, chloroquine (CQ, a late stage autophagy inhibitor that prevents fusion of autophagosomes with lysosomes) or rapamycin (an autophagy activator by inhibiting Akt/mTOR signal pathway) were applied to pharmacologically inhibit or accelerate the autophagy triggered by Rituximab-MMAE, respectively. In addition, two doses of ADC were applied to highlight the role of autophagy. Figure 5A showed that treatment with CQ led to an approximately 30% increase in the viability of Ramos and Daudi cells after Rituximab-MMAE exposure. On the contrary, Rituximab-MMAE-induced cytotoxicity against NHL cells was enhanced (Figure 5B; Figure S8 in Supplementary Material) when autophagy was further accelerated by rapamycin (Figures 5C,D; Figure S6C in Supplementary Material). The FCM study showed that treatment of Rituximab-MMAE with CQ reduced apoptotic cells, while combination of Rituximab-MMAE with rapamycin increased apoptosis induced by Rituximab-MMAE in NHL cells (Figures 5E,F). Besides, compared with cells treated with Rituximab-MMAE alone, cells treated with Rituximab-MMAE and rapamycin showed the increased cleavage of caspase 9 and PARP (Figures 5G,H; Figure S6D in Supplementary Material), while inhibition of apoptosis had no influence on the autophagy level in Rituximab-MMAE-treated NHL cells (Figure S9 in Supplementary Material). These data suggested that activation of autophagy further enhanced the apoptosis induced by Rituximab-MMAE.

Taken together, these results indicated that autophagy played a key role in the cytotoxicity of Rituximab-MMAE against Ramos and Daudi cells.

### Activation of Autophagy Enhanced Antitumor Efficacy of Rituximab-MMAE *In Vivo*

**Figure 6:**
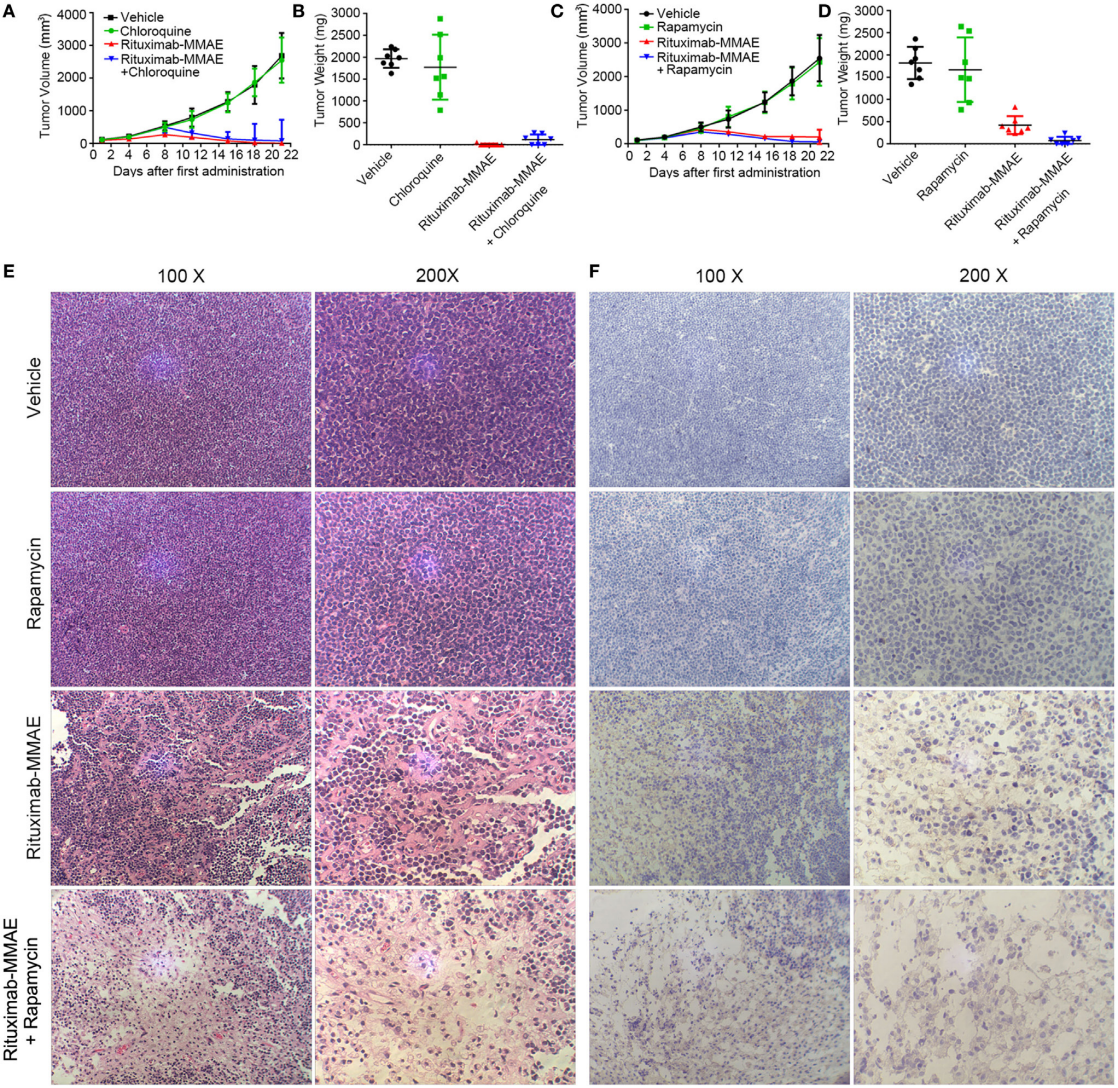
Activation of autophagy enhanced antitumor efficacy of Rituximab-monomethyl auristatin E (MMAE) *in vivo*. **(A–D)** BABL/c nude mice were transplanted subcutaneously with Ramos cells. Xenograft tumor volume was evaluated every other day by direct caliper measurements. There are seven mice in each group. The data were presented as means ± SD. Tumor weight was presented as mean ± SD, and each point represented a value from an independent mouse. **(E)** H&E staining images of Ramos xenograft tumors treated with Rituximab-MMAE with or without rapamycin. **(F)** Images of immunohistochemistry caspase 3 staining of Ramos xenograft tumors treated with Rituximab-MMAE with or without rapamycin.

Finally, we investigated whether targeting autophagy could be a potential therapeutic approach to enhance the anti-NHL efficacy of Rituximab-MMAE *in vivo*. Compared with the xenograft mice treated with Rituximab-MMAE alone, antitumor effects were attenuated in mice treated with both Rituximab-MMAE and chloroquine (Figure 6A). Tumor-bearing mice were sacrificed on day 22, and the tumor weight of mice treated Rituximab-MMAE (3 mg/kg, twice a week) alone was 7.14 ± 18.9 mg versus tumor weight of 1,822.86 ± 360.4 mg in the vehicle control. Importantly, six tumor-bearing mice were tumor free after 20 days of Rituximab-MMAE treatment, while the tumor weight in mice treated with Rituximab-MMAE in combination with chloroquine was 115.71 ± 119.84 mg and no tumor-bearing mice was tumor free after 20-day combination treatment (Figure 6B). On the other hand, tumor volume decreased significantly from day 8 in the mice co-treated with Rituximab-MMAE and rapamycin. After 20-day treatment, the mean tumor weight in mice treated with Rituximab-MMAE plus rapamycin, Rituximab-MMAE (0.75 mg/kg) and saline were 74.29 ± 87.91, 1,822.86 ± 360.4, and 422.86 ± 200.56 mg, respectively (Figures 6C,D; Figure S10 in Supplementary Material). Meanwhile, either chloroquine or rapamycin alone did not show obvious effect on the tumor growth in the xenograft models. Moreover, histopathologic analysis was applied to verify whether autophagy contributes to Rituximab-MMAE induced apoptosis *in vivo*. In tumor-bearing mice treated with both Rituximab-MMAE and rapamycin, tumor cell necrosis and caspase 3 expression increased, indicating that autophagy might contribute to the therapeutic efficacy of Rituximab-MMAE *in vivo* (Figures 6E,F).

Taken together, these results indicated that autophagy played a crucial role in the anti-NHL efficacy of Rituximab-MMAE and activating autophagy could significantly enhance the antitumor effect of Rituximab-MMAE *in vivo*.

## Discussion

A lot of efforts have been made in exploration of ADC for lymphoma therapies. The anti-CD30 ADC brentuximab vedotin was approved for therapy of Hodgkin lymphoma in 2011. Other ADC for NHL targeting CD19, CD79b, and CD22 was also in Phase I/II clinical trials (21–23). The choice of antigen target is one of the most important factors for a successful ADC. To reduce the off-target toxicity, the target antigen must be predominately expressed on the surface of target cells with minimal expression on healthy cells and could be internalized into the cell, so that ADC could be transported into the cell to exert its effect (24, 25). CD20, also named as human B-lymphocyte-restricted differentiation antigen, was expressed on most NHLs but rarely found on other normal tissues (26). ADC based on anti-CD20 antibody was used to be considered infeasible because of poor internalization and the only FDA approved conjugate based on anti-CD20 antibody is radiolabeled antibody which did not need to be internalized (27–30). Growing evidence suggested that type-1 anti-CD20 antibody, such as Rituximab and Ofatumumab, could be internalized intracellularly, which has provided new insight into the resistant mechanism of B cell malignancies (29, 31, 32). These studies made it possible to build an ADC based on anti-CD20 monoclonal antibody (27). In our study, the ADC based on anti-CD20 antibody has been generated and could be internalized in cells and showed potent antitumor efficacy.

Referred to as the first approved monoclonal antibody to treat NHLs, Rituximab is recognized as an ideal candidate for ADC development. Although previous study reported that Rituximab and auristatin-based ADC with the DAR ranging from 7.0 to 7.5 showed a potent antitumor efficacy *in vitro* and *in vivo* (33). Recent study showed that the DAR 4 conjugate was the optimal choice compared with the lower or higher DAR conjugates. ADC’s antitumor efficacy increased with the increase of DAR, hence a low drug load may weaken the antitumor effect of ADC (34). But DAR higher than four offered limited improvement in efficacy and excessive drug payload made ADC unstable and more prone to aggregation which significantly influenced the safety of ADC (35, 36). Therefore, the DAR 4 conjugate was the optimal choice in development of ADC. In this study, the ADC with optimal DAR of 4.2 was developed and demonstrated to have a powerful therapeutic efficacy and satisfactory safety profile both *in vitro* and *in vivo*.

To improve the safety and efficacy of ADCs, some new technologies have also been applied to the development. As the first bispecific T cell engager (BiTE) was approved by the FDA, ADC based on bispecific antibody which target two antigens, HER2 and CD63, was reported and may enhance lysosomal delivery and improve efficacy of ADCs (37, 38). On the other hand, installation of non-natural amino acid was applied to the new generation of ADC, the site-specific conjugation of the drug to engineered antibodies like THIOMAB leads to strictly controlled DARs (24, 37). Despite a lot of effort on the target and structures of ADCs has been done, the mechanism of ADC remains to be further studied.

To investigate the underlying mechanism of ADC, autophagy, an important mechanism in tumor therapy, has been increasingly regarded as a target for synergistic antitumor therapy (39, 40). In this study, we found that autophagy was activated in Rituximab-MMAE-treated NHL cells as evidenced by the accumulated autophagosomes, upregulated expression of LC3-II and LC3-II specific fluorescence. Furthermore, our data showed that inactivation of Akt/mTOR signal pathway was involved in the autophagy induced by Rituximab-MMAE, and autophagic flux including formation of autophagosomes, lysosomes fused with autophagosomes, and autophagosomes degradation by lysosomes was also observed in the Ramos cells and Daudi cells (Figure S11 in Supplementary Material). These results for the first time indicated that autophagy was involved in the treatment of cancer with ADC.

Growing evidence indicated that autophagy played a paradoxical role in the treatment of various cancer types during chemotherapy, radiotherapy, and immunotherapy (41–44). It was reported that doxorubicin and resveratrol induced excessive level of autophagy which resulted in cell death in MCF-7 breast cancer cells, while inhibiting of autophagy could impair their antitumor effect (45, 46). Furthermore, autophagy was also involved in the cytotoxicity induced by nanoparticles such as quantum dots and poly amidoamine dendrimers and their toxic effect could be attenuated by autophagy inhibitors (16–18). In this study, we found that autophagy and autophagic flux were activated by Rituximab-MMAE in NHL cells. Blocking the autophagy by autophagy inhibitor could suppress the antitumor efficacy of Rituximab-MMAE, while activating autophagy by rapamycin significantly enhanced the anti-NHL efficacy as evidenced by the increased apoptosis and activated apoptotic pathway.

**Figure 7:**
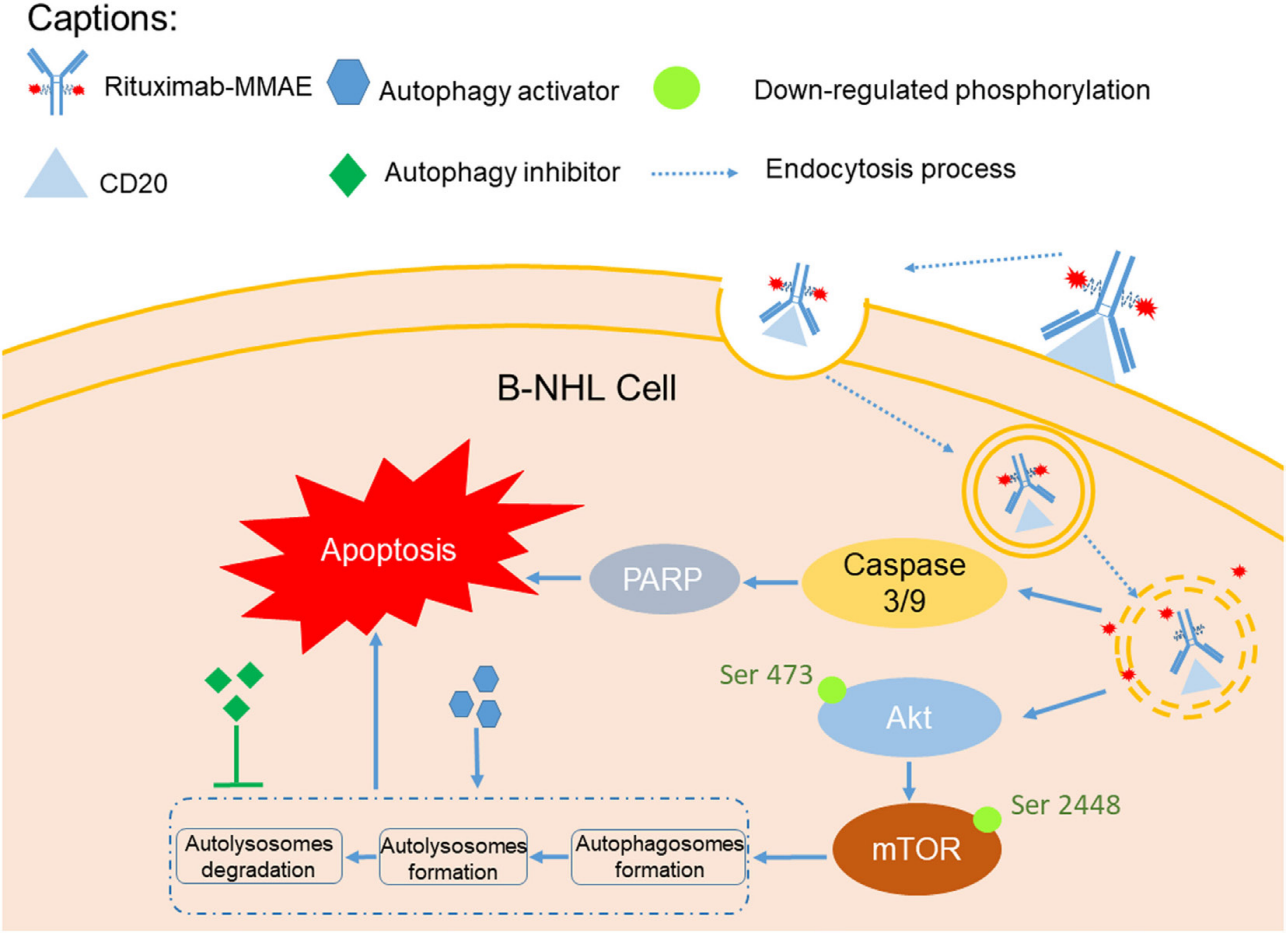
A graphical description of how Rituximab-monomethyl auristatin E (MMAE) and autophagy activation could elicit enhanced anti-non-Hodgkin lymphoma (NHL) effects.

In this study, we investigated the therapeutic efficacy of the novel ADC agent, Rituximab-MMAE, and evaluated the role of autophagy in the ADC-based NHL therapy (Figure 7). We demonstrated that Rituximab-MMAE could elicit potent antitumor efficacy *in vitro* and *in vivo*. Consist with earlier report about the ADC based on anti-CD30 antibody and MMAE, apoptosis was triggered by Rituximab-MMAE (19). More importantly, during this therapy, autophagy was triggered *via* inhibition of Akt/mTOR signal pathway, which was confirmed to play a cytotoxic role in the antitumor effects of ADC for the first time. Our data indicated that combination use of rapamycin with ADC could significantly enhance the therapeutic efficacy of Rituximab-MMAE *in vitro* and *in vivo* and highlighted the critical role of autophagy in ADC-based tumor therapy.

## Ethics Statement

This study was approved by Animal Ethical Committee of School of Pharmacy, Fudan University. Animal care and use were conducted according to the National Institutes of Health Guide for the Care and Use of Laboratory Animals (NIH publication No. 80-23, revised in 1996) after approval by Animal Ethical Committee of School of Pharmacy, Fudan University. Every effort was made to minimize animal suffering and number of animal used.

## Author Contributions

Experiment design: DJ, YWang, and XZ. Doing the experiment: YWang, XZ, JF, and YN. Data processing: WC, JL, SW, and QC. Paper writing: DJ, YZ, and YWu.

## Conflict of Interest Statement

The authors declare that the research was conducted in the absence of any commercial or financial relationships that could be construed as a potential conflict of interest. The reviewer YN and handling Editor declared their shared affiliation.
