# Supplementary material for: Activating Autophagy Enhanced the Antitumor Effect of Antibody Drug Conjugates Rituximab-Monomethyl Auristatin E
Source: Front Immunol. 2018 Aug 3;9:1799. doi: 10.3389/fimmu.2018.01799 (PMC6085421; doi:10.3389/fimmu.2018.01799)
Supplement: Supplementary file 4 [file data_sheet_4.PDF]

Activating Autophagy Enhanced the Antitumor Effect of Antibody Drug Conjugates Rituximab-MMAE

[*]**Corresponding author**: Dianwen Ju, Department of Microbiological and Biochemical Pharmacy & The Key Lab of Smart Drug Delivery, Ministry of Education, School of Pharmacy, Fudan University, Shanghai, 201203, P. R. China; E-mail: dianwenju@fudan.edu.cn; Tel: +86 21 51980037; Fax: +86 21 51980036.

Supplementary Data:

Supplementary Figure S1.

Supplementary Figure S2.

Supplementary Figure S3

Supplementary Figure S4

Supplementary Figure S5

Supplementary Figure S6

Supplementary Figure S7

Supplementary Figure S8

Supplementary Figure S9

Supplementary Figure S10

Supplementary Figure S11

Figure S4

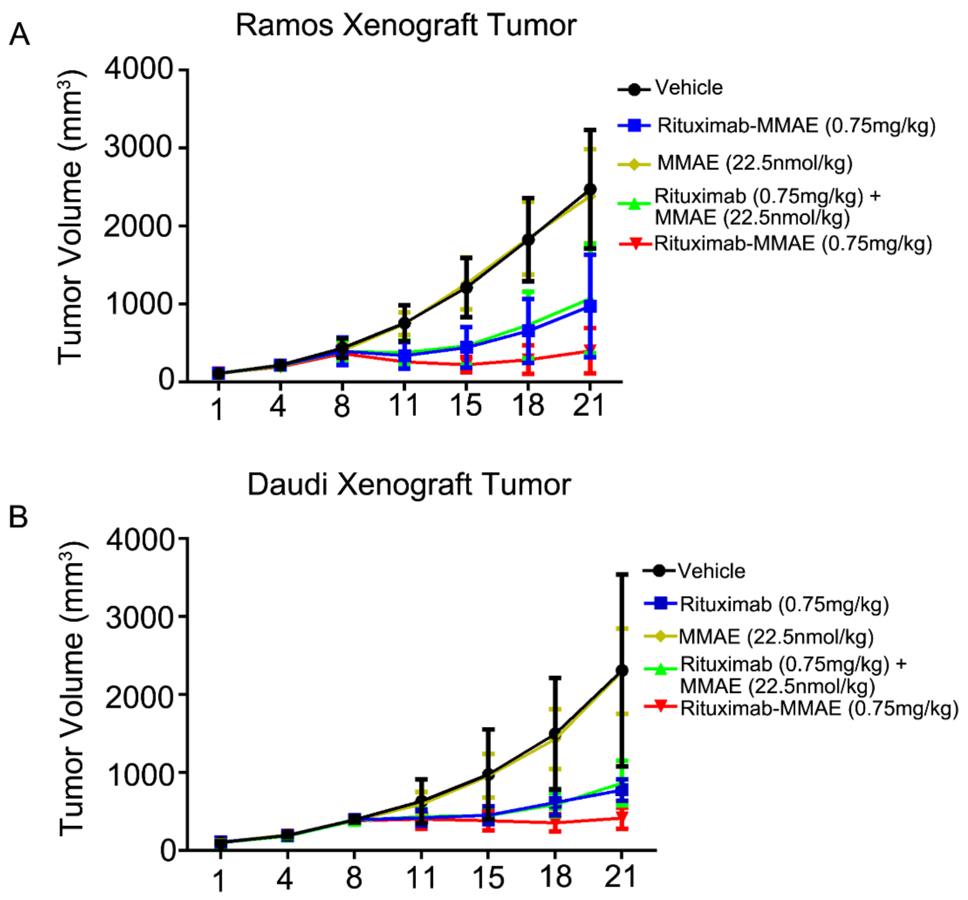

Figure S4. Growth curve of Ramos and Daudi xenograft tumor. (A) and (B) BABL/c nude mice were transplanted subcutaneously with Ramos cells and Daudi cells. There are 10 mice in each group. Mice was treated with Rituximab-MMAE (0.75 mg/kg), Rituximab (0.75 mg/kg), MMAE (22.5 nmol/kg) and Rituximab (0.75 mg/kg) + MMAE (22.5 nmol/kg) separately, twice a week for 21 days. Xenograft tumor volume was evaluated every other day by direct caliper measurements. The dose of MMAE was calculated from the dose of ADC. The data were presented as means ± SD.
