# Supplementary material for: Activating Autophagy Enhanced the Antitumor Effect of Antibody Drug Conjugates Rituximab-Monomethyl Auristatin E
Source: Front Immunol. 2018 Aug 3;9:1799. doi: 10.3389/fimmu.2018.01799 (PMC6085421; doi:10.3389/fimmu.2018.01799)
Supplement: Supplementary file 6 [file data_sheet_6.PDF]

Activating Autophagy Enhanced the Antitumor Effect of Antibody Drug Conjugates Rituximab-MMAE

*Corresponding author: Dianwen Ju, Department of Microbiological and Biochemical Pharmacy & The Key Lab of Smart Drug Delivery, Ministry of Education, School of Pharmacy, Fudan University, Shanghai, 201203, P. R. China; E-mail: dianwenju@fudan.edu.cn; Tel: +86 21 51980037; Fax: +86 21 51980036.

Supplementary Data:

Supplementary Figure S1.

Supplementary Figure S2.

Supplementary Figure S3

Supplementary Figure S4

Supplementary Figure S5

Supplementary Figure S6

Supplementary Figure S7

Supplementary Figure S8

Supplementary Figure S9

Supplementary Figure S10

Supplementary Figure S11

Figure S6

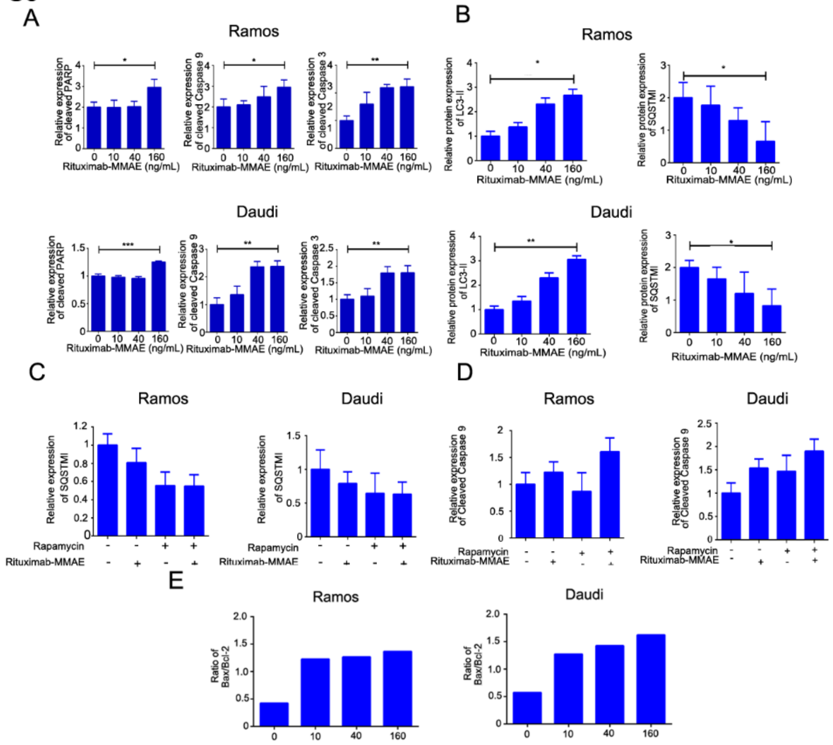

Figure S6. The statistical results of western blot in Figure 2, 3 and 5 (A) and (B) Densitometric values of apoptosis and autophagy related protein in B-NHL cells which exposed to different concentration of Rituximab-MMAE for 48 hours. (C) and (D) Densitometric values of SQSTM1 and Cleaved Caspase 9 in B-NHL cells treated with or without Rituximab-MMAE and Rapamycin. (E) Bar diagram summarizing the effects of Rituximab-MMAE treatment on Bax/Bcl-2 ratio.
