# Supplementary material for: Activating Autophagy Enhanced the Antitumor Effect of Antibody Drug Conjugates Rituximab-Monomethyl Auristatin E
Source: Front Immunol. 2018 Aug 3;9:1799. doi: 10.3389/fimmu.2018.01799 (PMC6085421; doi:10.3389/fimmu.2018.01799)
Supplement: Supplementary file 9 [file data_sheet_9.PDF]

Activating Autophagy Enhanced the Antitumor Effect of Antibody Drug Conjugates Rituximab-MMAE

*Corresponding author: Dianwen Ju, Department of Microbiological and Biochemical Pharmacy & The Key Lab of Smart Drug Delivery, Ministry of Education, School of Pharmacy, Fudan University, Shanghai, 201203, P. R. China; E-mail: dianwenju@fudan.edu.cn; Tel: +86 21 51980037; Fax: +86 21 51980036.

Supplementary Data:

Supplementary Figure S1.

Supplementary Figure S2.

Supplementary Figure S3

Supplementary Figure S4

Supplementary Figure S5

Supplementary Figure S6

Supplementary Figure S7

Supplementary Figure S8

Supplementary Figure S9

Supplementary Figure S10

Supplementary Figure S11

Figure S9

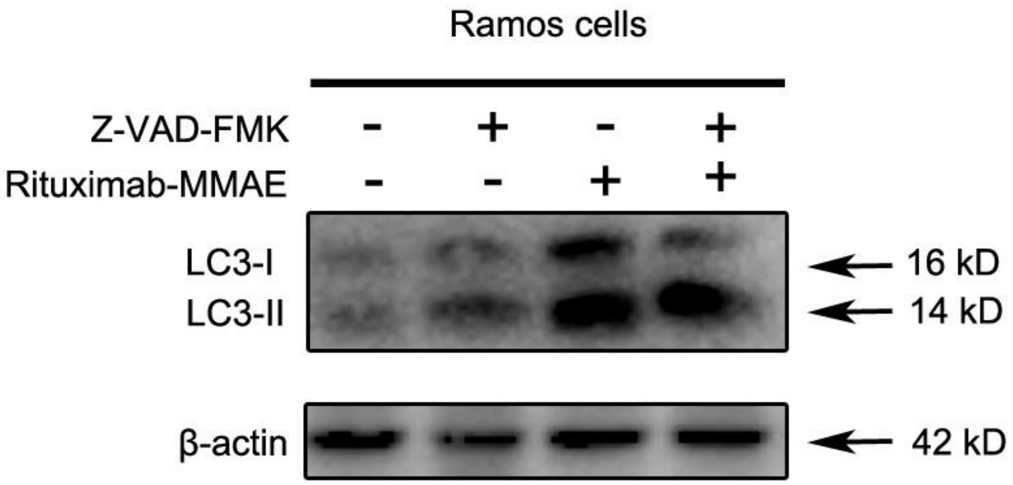

Figure S9. Ramos cells were treated with Rituximab-MMAE (40 ng/mL) combined with the pan-caspase inhibitor Z-VAD-FMK (50 μM) for 48h, protein expressions of LC3-I and II were measured by western blot.
