# Supplementary material for: Activating Autophagy Enhanced the Antitumor Effect of Antibody Drug Conjugates Rituximab-Monomethyl Auristatin E
Source: Front Immunol. 2018 Aug 3;9:1799. doi: 10.3389/fimmu.2018.01799 (PMC6085421; doi:10.3389/fimmu.2018.01799)
Supplement: Supplementary file 10 [file data_sheet_10.PDF]

Activating Autophagy Enhanced the Antitumor Effect of Antibody Drug Conjugates Rituximab-MMAE

*Corresponding author: Dianwen Ju, Department of Microbiological and Biochemical Pharmacy & The Key Lab of Smart Drug Delivery, Ministry of Education, School of Pharmacy, Fudan University, Shanghai, 201203, P. R. China; E-mail: dianwenju@fudan.edu.cn; Tel: +86 21 51980037; Fax: +86 21 51980036.

Supplementary Data:

Supplementary Figure S1.

Supplementary Figure S2.

Supplementary Figure S3

Supplementary Figure S4

Supplementary Figure S5

Supplementary Figure S6

Supplementary Figure S7

Supplementary Figure S8

Supplementary Figure S9

Supplementary Figure S10

Supplementary Figure S11

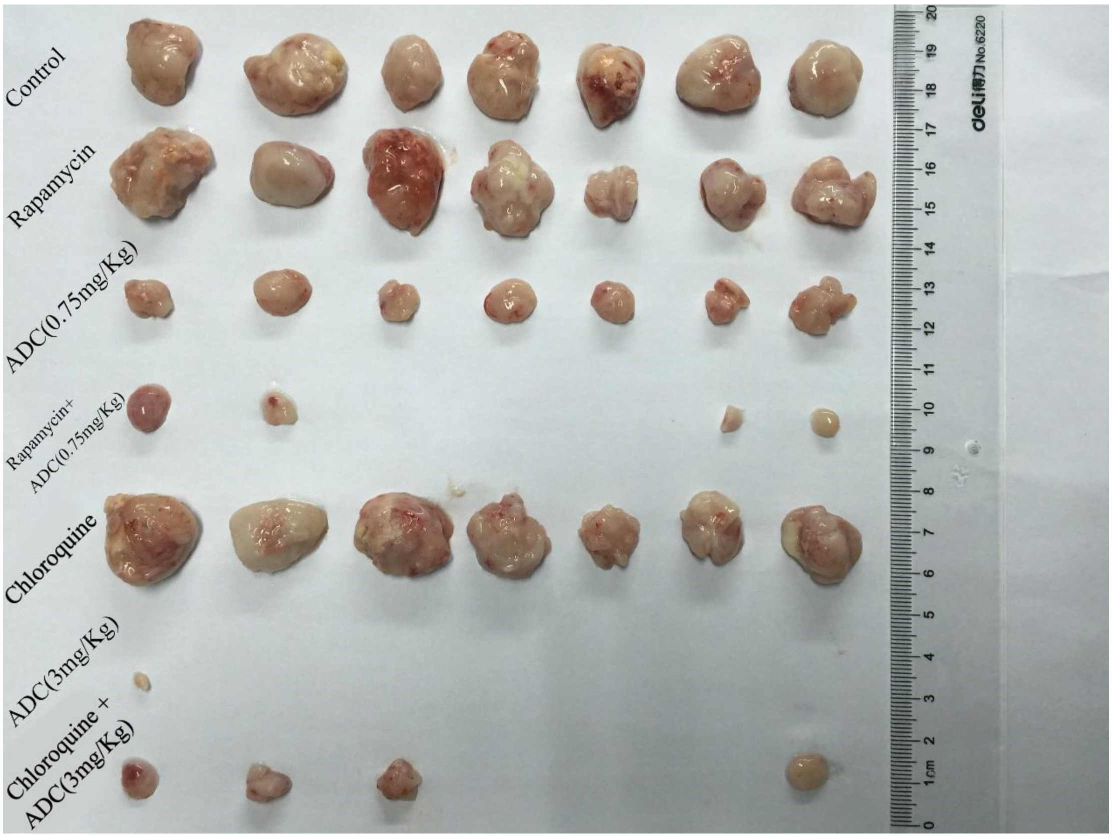

Figure S10. The restricted Ramos xenograft tumors after treatment with Rituximab-MMAE and/or chloroquine and rapamycin. Tumor free rate for each group: Control: 0%; Rapamycin: 0%; Rituximab-MMAE (0.75mg/kg): 0%; Rapamycin + Rituximab-MMAE (0.75mg/kg):42.9%; Chloroquine: 0%; Rituximab-MMAE (3mg/kg): 85.7%; Chloroquine + Rituximab-MMAE (3mg/kg): 42.9%.
